# Bis{tris[2-(2-oxidobenzylideneazaniumyl)ethyl]amine-κ^3^ *O*,*O*′,*O*′′}calcium bis(perchlorate) acetonitrile disolvate

**DOI:** 10.1107/S1600536810053961

**Published:** 2011-01-08

**Authors:** Muhammet Kose, Vickie McKee

**Affiliations:** a Chemistry Department, Loughborough University, Leicestershire, LE11 3TU, England

## Abstract

The title complex, [Ca(C_27_H_30_N_4_O_3_)_2_](ClO_4_)_2_·2CH_3_CN, is composed of centrosymmetric (Ca*L_2_*)^2+^ cations [*L* = tris­(2-hy­droxy­benzoyl­amino­eth­yl)amine = H_3_saltren], uncoordin­ated perchlorate anions and acetonitrile solvent mol­ecules. The calcium ion is six-coordinated and is bonded to all phen­oxy O atoms from both zwitterionic saltren mol­ecules. There are strong intra­molecular N—H⋯O hydrogen bonds. The cations are linked into chains *via* weak inter­molecular C—H⋯O hydrogen bonds and C—H⋯π and π–π stacking inter­actions [centroid–centroid distances = 3.306 (3) and 3.415 (3) Å].

## Related literature

For crystal structure of the free ligand, see: Gündüz *et al.* (1985 ▶). For structures of transition metal complexes of H_3_saltren, see: Steinhauser *et al.* (2004 ▶); Elerman *et al.* (1994 ▶, 1995 ▶).
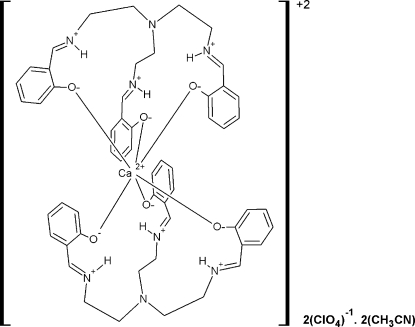


## Experimental

### 

#### Crystal data

[Ca(C_27_H_30_N_4_O_3_)_2_](ClO_4_)_2_·2C_2_H_3_N
*M* *_r_* = 1238.19
Orthorhombic, 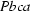

*a* = 11.3469 (7) Å
*b* = 19.5307 (12) Å
*c* = 27.3178 (16) Å
*V* = 6054.0 (6) Å^3^
*Z* = 4
Mo *K*α radiation
μ = 0.27 mm^−1^
*T* = 150 K
0.12 × 0.10 × 0.09 mm

#### Data collection

Bruker APEXII CCD diffractometer
Absorption correction: multi-scan (*SADABS*; Bruker, 2008 ▶) *T*_min_ = 0.662, *T*_max_ = 0.746
55467 measured reflections
6915 independent reflections
5810 reflections with *I* > 2.0σ(*I*)
*R*_int_ = 0.030

#### Refinement

*R*[*F* ^2^ > 2σ(*F* ^2^)] = 0.039
*wR*(*F* ^2^) = 0.110
*S* = 1.05
6915 reflections
398 parameters
H atoms treated by a mixture of independent and constrained refinement
Δρ_max_ = 0.60 e Å^−3^
Δρ_min_ = −0.39 e Å^−3^

### 

Data collection: *APEX2* (Bruker, 2008 ▶); cell refinement: *SAINT* (Bruker, 2008 ▶); data reduction: *SAINT*; program(s) used to solve structure: *SHELXS97* (Sheldrick, 2008 ▶); program(s) used to refine structure: *SHELXL97* (Sheldrick, 2008 ▶); molecular graphics: *SHELXTL* (Sheldrick, 2008 ▶); software used to prepare material for publication: *SHELXTL*.

## Supplementary Material

Crystal structure: contains datablocks I, global. DOI: 10.1107/S1600536810053961/pv2371sup1.cif

Structure factors: contains datablocks I. DOI: 10.1107/S1600536810053961/pv2371Isup2.hkl

Additional supplementary materials:  crystallographic information; 3D view; checkCIF report

## Figures and Tables

**Table 1:** Hydrogen-bond geometry (Å, °) *Cg*1 is the centroid of the C1–C6 ring.

*D*—H⋯*A*	*D*—H	H⋯*A*	*D*⋯*A*	*D*—H⋯*A*
N1—H1*A*⋯O1	0.85 (2)	1.98 (2)	2.6597 (17)	136 (2)
N3—H3*A*⋯O2	0.86 (2)	1.91 (2)	2.6256 (18)	140 (2)
N4—H4*A*⋯O3	0.83 (2)	1.98 (2)	2.6449 (17)	137 (2)
C3—H3⋯O7^i^	0.95	2.41	3.316 (2)	160
C20—H20*A*⋯O5^ii^	0.99	2.50	3.380 (2)	148
C10—H10*A*⋯O5^iii^	0.99	2.50	3.271 (2)	134
C21—H21⋯O5^ii^	0.95	2.58	3.259 (2)	129
C29—H29*B*⋯O6^iv^	0.98	2.58	3.555 (3)	173
C26—H26⋯*Cg*1^v^	0.95	2.68	3.489 (2)	143

Symmetry codes: (i) 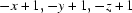; (ii) 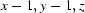; (iii) 
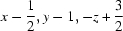
; (iv) 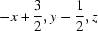; (v) 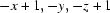.

**Fig. 1.**
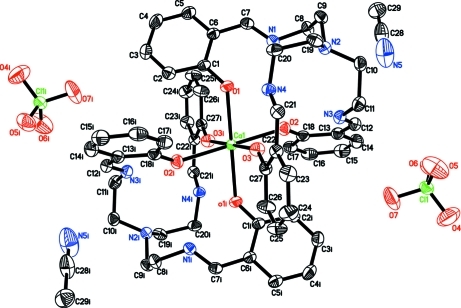
Perspective view of centrosymmetric calcium complex [Ca(C27H30N4O3)2](ClO4)2(CH3CN)2; displacement ellipsoids are drawn at the 50% probability level and hydrogen atoms are omitted for clarity (Symmetry code (i) -x + 1, -y, -z + 1).

**Fig. 2.**
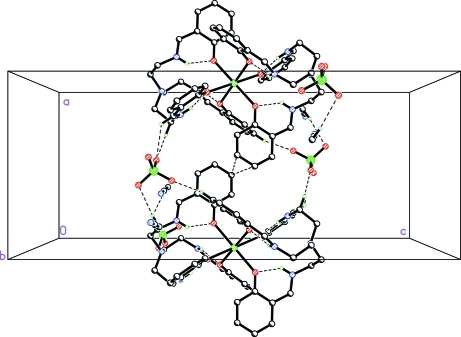
Packing diagram viewed perpendicular to b, showing intramolecular hydrogen bonds and π-π (phenyl-phenyl) interactions.

## supplementary crystallographic information

### Comment

The crystal structures and coordination chemistry of transition metal complexes of H_3_saltren, tris(2-hydroxybenzoylaminoethyl)amine (*L*), a tripodal ligand, have been described in detail (Steinhauser *et al.*, 2004; Elerman *et al.*, 1994); Elerman *et al.*, 1995). Here, we report the first example of an earth alkaline metal (Ca^+2^) complex of H_3_saltren.

The crystal structure of the title complex contains centrosymmetric (Ca*L_2_*)^+2^ cations, uncoordinated perchlorate anions and acetonitrile molecules (Fig. 1). Each calcium ion is six-coordinated, bonded to all phenoxy O atoms from two saltren molecules. There are strong intramolecular hydrogen bonds of the type N—H···O between protonated imine and deprotonated phenol-oxygen atoms (Table 1). The cations are linked into chains *via* intermolecular C—H···O and C—H··· N type weak hydrogen bonds and C—H···π interactions (Tab. 1 and Fig. 2). There is evidence of π-π stacking in the crystal structure; C16 is stacked with C16^*^ and C15^*^ of an adjacent molecule (^*^ = 2 - *x*, -*y*, 1 - *z*) with separation of 3.306 (3) and 3.415 (3) Å, respectively (Fig. 2). Protonated imine distances (C═N distances) (mean 1.300 (2) Å) are longer than the corresponding C═N distances reported in the neutral ligand (mean 1.267 Å) (Gündüz *et al.*, 1985). The longer C═N distances were also determined by IR spectroscopy; while C═N stretch is observed at 1633 cm^-1 ^ in the neutral ligand, by protonation of imine, C═N stretch shifts to 1654 cm^-1^.

### Experimental

H_3_Saltren (0.508 g, 1.11 mmol) was dissolved in methanol (50 ml) followed by addition of Ca(ClO_4_)_2_.3H_2_O (0.185 g, 0.63 mmol) in methanol (20 ml). The reaction mixture was stirred at room temperature for two hours. Yellow precipitate was collected and washed with methanol (5 ml) and diethylether (20 ml) (yield 0.506 g, 0.44 mmol, 69.6%). X-ray quality crystals were obtained from acetonitrile solution of the title complex by slow evaporation.

### Refinement

H atoms bonded to C were inserted at calculated positions with C—H distances of 0.95, 0.98 and 0.99 Å for aryl, methyl and methylene H atoms, respectively, and refined using a riding model with *U*_iso_(H) = 1.2 or 1.5*U*_eq_(C). The H-atoms bonded to N-atoms were taken from the difference Fourier maps and were refined isotropically.

### Crystal data

**Table:**

[Ca(C_27_H_30_N_4_O_3_)_2_](ClO_4_)_2_·2C_2_H_3_N	*F*(000) = 2600
*M**_r_* = 1238.19	*D*_x_ = 1.358 Mg m^−^^3^
Orthorhombic, *P**b**c**a*	Mo *K*α radiation, λ = 0.71073 Å
Hall symbol: -P 2ac 2ab	Cell parameters from 9950 reflections
*a* = 11.3469 (7) Å	θ = 2.2–27.4°
*b* = 19.5307 (12) Å	µ = 0.27 mm^−^^1^
*c* = 27.3178 (16) Å	*T* = 150 K
*V* = 6054.0 (6) Å^3^	Block, yellow
*Z* = 4	0.12 × 0.10 × 0.09 mm

### Data collection

**Table:**

Bruker APEXII CCD diffractometer	6915 independent reflections
Radiation source: fine-focus sealed tube	5810 reflections with *I* > 2.0σ(*I*)
graphite	*R*_int_ = 0.030
φ and ω scans	θ_max_ = 27.5°, θ_min_ = 1.5°
Absorption correction: multi-scan (*SADABS*; Bruker, 2008)	*h* = −14→14
*T*_min_ = 0.662, *T*_max_ = 0.746	*k* = −25→25
55467 measured reflections	*l* = −34→35

### Refinement

**Table:**

Refinement on *F*^2^	Primary atom site location: structure-invariant direct methods
Least-squares matrix: full	Secondary atom site location: difference Fourier map
*R*[*F*^2^ > 2σ(*F*^2^)] = 0.039	Hydrogen site location: inferred from neighbouring sites
*wR*(*F*^2^) = 0.110	H atoms treated by a mixture of independent and constrained refinement
*S* = 1.05	*w* = 1/[σ^2^(*F*_o_^2^) + (0.0538*P*)^2^ + 3.4246*P*] where *P* = (*F*_o_^2^ + 2*F*_c_^2^)/3
6915 reflections	(Δ/σ)_max_ = 0.002
398 parameters	Δρ_max_ = 0.60 e Å^−^^3^
0 restraints	Δρ_min_ = −0.39 e Å^−^^3^

### Special details

**Table:**

Geometry. All e.s.d.'s (except the e.s.d. in the dihedral angle between two l.s. planes) are estimated using the full covariance matrix. The cell e.s.d.'s are taken into account individually in the estimation of e.s.d.'s in distances, angles and torsion angles; correlations between e.s.d.'s in cell parameters are only used when they are defined by crystal symmetry. An approximate (isotropic) treatment of cell e.s.d.'s is used for estimating e.s.d.'s involving l.s. planes.
Refinement. Refinement of *F*^2^ against ALL reflections. The weighted *R*-factor *wR* and goodness of fit *S* are based on *F*^2^, conventional *R*-factors *R* are based on *F*, with *F* set to zero for negative *F*^2^. The threshold expression of *F*^2^ > σ(*F*^2^) is used only for calculating *R*-factors(gt) *etc*. and is not relevant to the choice of reflections for refinement. *R*-factors based on *F*^2^ are statistically about twice as large as those based on *F*, and *R*- factors based on ALL data will be even larger.

### Fractional atomic coordinates and isotropic or equivalent isotropic displacement parameters (Å^2^)

**Table:**

	*x*	*y*	*z*	*U*_iso_*/*U*_eq_	
Ca1	0.5000	0.0000	0.5000	0.01901 (10)	
O1	0.38283 (10)	0.08611 (5)	0.53706 (4)	0.0260 (2)	
C1	0.32707 (13)	0.13534 (8)	0.51499 (5)	0.0224 (3)	
C2	0.25547 (15)	0.12409 (8)	0.47327 (6)	0.0291 (3)	
H2	0.2438	0.0786	0.4619	0.035*	
C3	0.20240 (16)	0.17746 (10)	0.44891 (7)	0.0349 (4)	
H3	0.1557	0.1680	0.4208	0.042*	
C4	0.21547 (17)	0.24547 (9)	0.46452 (7)	0.0395 (4)	
H4	0.1795	0.2818	0.4469	0.047*	
C5	0.28043 (16)	0.25839 (9)	0.50526 (7)	0.0340 (4)	
H5	0.2879	0.3042	0.5166	0.041*	
C6	0.33725 (13)	0.20497 (8)	0.53117 (6)	0.0239 (3)	
C7	0.40674 (14)	0.22286 (8)	0.57213 (5)	0.0247 (3)	
H7	0.4066	0.2695	0.5821	0.030*	
N1	0.47075 (12)	0.18036 (7)	0.59710 (5)	0.0243 (3)	
C8	0.53399 (15)	0.19940 (8)	0.64171 (6)	0.0277 (3)	
H8A	0.5444	0.2497	0.6429	0.033*	
H8B	0.6130	0.1779	0.6418	0.033*	
C9	0.46415 (15)	0.17550 (8)	0.68610 (6)	0.0264 (3)	
H9A	0.5005	0.1939	0.7163	0.032*	
H9B	0.3825	0.1931	0.6840	0.032*	
N2	0.46228 (11)	0.10039 (6)	0.68827 (5)	0.0228 (3)	
C10	0.56302 (15)	0.07362 (9)	0.71617 (6)	0.0290 (3)	
H10A	0.5388	0.0659	0.7505	0.035*	
H10B	0.6271	0.1080	0.7162	0.035*	
C11	0.60888 (15)	0.00683 (9)	0.69464 (6)	0.0307 (4)	
H11A	0.6682	−0.0133	0.7170	0.037*	
H11B	0.5431	−0.0261	0.6913	0.037*	
N3	0.66223 (12)	0.01909 (7)	0.64666 (5)	0.0283 (3)	
C12	0.77320 (14)	0.03357 (9)	0.64084 (6)	0.0303 (3)	
H12	0.8224	0.0329	0.6690	0.036*	
C13	0.82570 (14)	0.05027 (8)	0.59528 (6)	0.0282 (3)	
C14	0.94893 (15)	0.06200 (9)	0.59374 (7)	0.0362 (4)	
H14	0.9933	0.0605	0.6232	0.043*	
C15	1.00481 (16)	0.07539 (9)	0.55058 (8)	0.0385 (4)	
H15	1.0872	0.0841	0.5499	0.046*	
C16	0.93896 (16)	0.07617 (9)	0.50724 (7)	0.0353 (4)	
H16	0.9780	0.0844	0.4770	0.042*	
C17	0.81902 (16)	0.06529 (8)	0.50726 (6)	0.0313 (4)	
H17	0.7772	0.0660	0.4771	0.038*	
C18	0.75700 (14)	0.05306 (7)	0.55146 (6)	0.0253 (3)	
O2	0.64376 (10)	0.04299 (6)	0.55239 (4)	0.0287 (2)	
C19	0.34878 (15)	0.07321 (8)	0.70527 (6)	0.0280 (3)	
H19A	0.3107	0.1072	0.7270	0.034*	
H19B	0.3626	0.0311	0.7246	0.034*	
C20	0.26672 (14)	0.05700 (8)	0.66278 (6)	0.0287 (3)	
H20A	0.1863	0.0484	0.6754	0.034*	
H20B	0.2630	0.0968	0.6404	0.034*	
N4	0.30786 (12)	−0.00316 (6)	0.63575 (5)	0.0241 (3)	
C21	0.29327 (13)	−0.06510 (8)	0.65229 (6)	0.0252 (3)	
H21	0.2469	−0.0706	0.6810	0.030*	
C22	0.34110 (13)	−0.12487 (8)	0.63089 (6)	0.0233 (3)	
C23	0.31923 (15)	−0.18869 (8)	0.65401 (6)	0.0308 (4)	
H23	0.2708	−0.1903	0.6824	0.037*	
C24	0.36664 (16)	−0.24768 (9)	0.63612 (7)	0.0361 (4)	
H24	0.3526	−0.2901	0.6521	0.043*	
C25	0.43665 (16)	−0.24480 (9)	0.59373 (7)	0.0376 (4)	
H25	0.4690	−0.2859	0.5809	0.045*	
C26	0.45932 (16)	−0.18409 (9)	0.57042 (7)	0.0341 (4)	
H26	0.5066	−0.1842	0.5417	0.041*	
C27	0.41389 (13)	−0.12088 (8)	0.58811 (5)	0.0231 (3)	
O3	0.43711 (10)	−0.06298 (6)	0.56747 (4)	0.0285 (3)	
C28	0.8355 (2)	0.24381 (14)	0.68889 (8)	0.0537 (6)	
C29	0.85639 (19)	0.31612 (11)	0.68130 (9)	0.0505 (5)	
H29A	0.9341	0.3283	0.6943	0.076*	
H29B	0.7955	0.3427	0.6982	0.076*	
H29C	0.8538	0.3263	0.6462	0.076*	
N5	0.8201 (3)	0.18674 (14)	0.69423 (9)	0.0870 (9)	
H4A	0.3474 (18)	−0.0002 (10)	0.6103 (8)	0.034 (5)*	
H3A	0.6222 (19)	0.0209 (11)	0.6199 (8)	0.040 (6)*	
H1A	0.4716 (19)	0.1395 (12)	0.5863 (8)	0.046 (6)*	
Cl1	0.96706 (4)	0.89879 (2)	0.698669 (15)	0.03209 (11)	
O4	1.04850 (18)	0.84563 (10)	0.70855 (9)	0.0828 (6)	
O5	1.02963 (14)	0.96257 (8)	0.69527 (6)	0.0547 (4)	
O6	0.88221 (16)	0.90508 (10)	0.73665 (7)	0.0743 (6)	
O7	0.90949 (18)	0.88552 (10)	0.65378 (6)	0.0699 (5)	

### Atomic displacement parameters (Å^2^)

**Table:**

	*U*^11^	*U*^22^	*U*^33^	*U*^12^	*U*^13^	*U*^23^
Ca1	0.0219 (2)	0.01807 (19)	0.01709 (19)	0.00034 (15)	0.00046 (15)	0.00076 (14)
O1	0.0295 (6)	0.0200 (5)	0.0285 (6)	0.0045 (4)	−0.0004 (4)	0.0005 (4)
C1	0.0202 (7)	0.0226 (7)	0.0243 (7)	0.0021 (6)	0.0052 (6)	0.0012 (6)
C2	0.0294 (8)	0.0270 (8)	0.0309 (8)	0.0006 (6)	−0.0015 (7)	−0.0034 (6)
C3	0.0317 (9)	0.0410 (10)	0.0321 (9)	0.0026 (7)	−0.0085 (7)	0.0006 (7)
C4	0.0426 (10)	0.0326 (9)	0.0432 (10)	0.0087 (8)	−0.0118 (8)	0.0084 (8)
C5	0.0383 (9)	0.0225 (8)	0.0413 (10)	0.0049 (7)	−0.0063 (7)	0.0016 (7)
C6	0.0253 (7)	0.0219 (7)	0.0245 (7)	0.0019 (6)	0.0014 (6)	0.0013 (6)
C7	0.0284 (8)	0.0207 (7)	0.0251 (7)	−0.0012 (6)	0.0041 (6)	0.0001 (6)
N1	0.0290 (7)	0.0219 (6)	0.0219 (6)	−0.0040 (5)	0.0004 (5)	0.0009 (5)
C8	0.0337 (8)	0.0272 (8)	0.0222 (7)	−0.0086 (6)	−0.0027 (6)	0.0023 (6)
C9	0.0342 (8)	0.0238 (7)	0.0211 (7)	−0.0018 (6)	0.0006 (6)	−0.0010 (6)
N2	0.0250 (6)	0.0220 (6)	0.0214 (6)	−0.0021 (5)	0.0007 (5)	0.0024 (5)
C10	0.0324 (9)	0.0321 (8)	0.0224 (7)	0.0001 (7)	−0.0039 (6)	0.0022 (6)
C11	0.0312 (8)	0.0319 (8)	0.0290 (8)	0.0031 (7)	−0.0017 (7)	0.0065 (6)
N3	0.0261 (7)	0.0308 (7)	0.0279 (7)	0.0026 (6)	−0.0029 (6)	0.0001 (6)
C12	0.0266 (8)	0.0315 (8)	0.0326 (8)	0.0039 (6)	−0.0062 (6)	−0.0042 (7)
C13	0.0246 (8)	0.0255 (7)	0.0344 (8)	0.0007 (6)	−0.0018 (6)	−0.0045 (6)
C14	0.0257 (8)	0.0388 (10)	0.0442 (10)	−0.0014 (7)	−0.0047 (7)	−0.0077 (8)
C15	0.0254 (8)	0.0340 (9)	0.0561 (12)	−0.0043 (7)	0.0050 (8)	−0.0066 (8)
C16	0.0367 (9)	0.0250 (8)	0.0442 (10)	−0.0025 (7)	0.0113 (8)	−0.0018 (7)
C17	0.0355 (9)	0.0232 (8)	0.0352 (9)	−0.0019 (6)	0.0016 (7)	0.0001 (6)
C18	0.0266 (7)	0.0164 (7)	0.0330 (8)	−0.0008 (6)	−0.0011 (6)	−0.0029 (6)
O2	0.0248 (6)	0.0308 (6)	0.0304 (6)	−0.0023 (4)	−0.0034 (4)	−0.0008 (5)
C19	0.0309 (8)	0.0266 (8)	0.0265 (8)	−0.0046 (6)	0.0083 (6)	−0.0034 (6)
C20	0.0237 (8)	0.0246 (8)	0.0377 (9)	0.0013 (6)	0.0047 (6)	−0.0053 (6)
N4	0.0224 (6)	0.0230 (6)	0.0269 (7)	−0.0008 (5)	0.0023 (5)	−0.0025 (5)
C21	0.0216 (7)	0.0280 (8)	0.0262 (7)	−0.0026 (6)	0.0026 (6)	−0.0006 (6)
C22	0.0226 (7)	0.0227 (7)	0.0245 (7)	−0.0018 (6)	0.0009 (6)	0.0010 (6)
C23	0.0315 (8)	0.0283 (8)	0.0328 (9)	−0.0072 (6)	0.0058 (7)	0.0050 (7)
C24	0.0385 (9)	0.0211 (8)	0.0486 (10)	−0.0063 (7)	0.0035 (8)	0.0059 (7)
C25	0.0402 (10)	0.0217 (8)	0.0509 (11)	−0.0003 (7)	0.0077 (8)	−0.0063 (7)
C26	0.0387 (9)	0.0281 (8)	0.0356 (9)	−0.0017 (7)	0.0125 (7)	−0.0049 (7)
C27	0.0241 (7)	0.0236 (7)	0.0217 (7)	−0.0019 (6)	−0.0004 (6)	0.0009 (6)
O3	0.0356 (6)	0.0242 (6)	0.0257 (6)	−0.0002 (5)	0.0054 (5)	0.0050 (4)
C28	0.0484 (12)	0.0695 (15)	0.0431 (11)	−0.0228 (11)	−0.0127 (9)	0.0133 (10)
C29	0.0410 (11)	0.0524 (13)	0.0582 (13)	0.0026 (9)	0.0053 (10)	0.0008 (10)
N5	0.105 (2)	0.0817 (17)	0.0743 (16)	−0.0495 (15)	−0.0320 (14)	0.0278 (13)
Cl1	0.0298 (2)	0.0318 (2)	0.0346 (2)	−0.00051 (15)	−0.00320 (16)	−0.00806 (16)
O4	0.0778 (13)	0.0586 (11)	0.1119 (17)	0.0288 (10)	−0.0151 (12)	0.0028 (11)
O5	0.0497 (9)	0.0468 (9)	0.0675 (10)	−0.0183 (7)	0.0038 (7)	−0.0143 (7)
O6	0.0635 (11)	0.0894 (13)	0.0702 (12)	−0.0249 (10)	0.0329 (9)	−0.0277 (10)
O7	0.0905 (13)	0.0724 (11)	0.0469 (9)	−0.0234 (10)	−0.0246 (9)	−0.0098 (8)

### Geometric parameters (Å, °)

**Table:**

Ca1—O2^i^	2.3269 (11)	C13—C18	1.430 (2)
Ca1—O2	2.3269 (11)	C14—C15	1.364 (3)
Ca1—O3^i^	2.3279 (11)	C14—H14	0.9500
Ca1—O3	2.3279 (11)	C15—C16	1.400 (3)
Ca1—O1^i^	2.3709 (10)	C15—H15	0.9500
Ca1—O1	2.3709 (10)	C16—C17	1.377 (3)
O1—C1	1.2993 (18)	C16—H16	0.9500
C1—C2	1.417 (2)	C17—C18	1.418 (2)
C1—C6	1.435 (2)	C17—H17	0.9500
C2—C3	1.375 (2)	C18—O2	1.3001 (19)
C2—H2	0.9500	C19—C20	1.522 (2)
C3—C4	1.403 (3)	C19—H19A	0.9900
C3—H3	0.9500	C19—H19B	0.9900
C4—C5	1.358 (3)	C20—N4	1.4642 (19)
C4—H4	0.9500	C20—H20A	0.9900
C5—C6	1.416 (2)	C20—H20B	0.9900
C5—H5	0.9500	N4—C21	1.302 (2)
C6—C7	1.413 (2)	N4—H4A	0.83 (2)
C7—N1	1.297 (2)	C21—C22	1.414 (2)
C7—H7	0.9500	C21—H21	0.9500
N1—C8	1.462 (2)	C22—C23	1.419 (2)
N1—H1A	0.85 (2)	C22—C27	1.433 (2)
C8—C9	1.522 (2)	C23—C24	1.362 (2)
C8—H8A	0.9900	C23—H23	0.9500
C8—H8B	0.9900	C24—C25	1.405 (3)
C9—N2	1.4684 (19)	C24—H24	0.9500
C9—H9A	0.9900	C25—C26	1.370 (2)
C9—H9B	0.9900	C25—H25	0.9500
N2—C19	1.468 (2)	C26—C27	1.422 (2)
N2—C10	1.470 (2)	C26—H26	0.9500
C10—C11	1.523 (2)	C27—O3	1.2908 (18)
C10—H10A	0.9900	C28—N5	1.138 (3)
C10—H10B	0.9900	C28—C29	1.447 (3)
C11—N3	1.463 (2)	C29—H29A	0.9800
C11—H11A	0.9900	C29—H29B	0.9800
C11—H11B	0.9900	C29—H29C	0.9800
N3—C12	1.300 (2)	Cl1—O7	1.4135 (16)
N3—H3A	0.86 (2)	Cl1—O4	1.4158 (18)
C12—C13	1.418 (2)	Cl1—O6	1.4209 (16)
C12—H12	0.9500	Cl1—O5	1.4368 (15)
C13—C14	1.418 (2)		
			
O2^i^—Ca1—O2	180.00 (4)	N3—C12—H12	117.8
O2^i^—Ca1—O3^i^	85.33 (4)	C13—C12—H12	117.8
O2—Ca1—O3^i^	94.67 (4)	C14—C13—C12	118.54 (16)
O2^i^—Ca1—O3	94.67 (4)	C14—C13—C18	120.45 (16)
O2—Ca1—O3	85.33 (4)	C12—C13—C18	120.98 (14)
O3^i^—Ca1—O3	180.0	C15—C14—C13	121.02 (17)
O2^i^—Ca1—O1^i^	82.79 (4)	C15—C14—H14	119.5
O2—Ca1—O1^i^	97.21 (4)	C13—C14—H14	119.5
O3^i^—Ca1—O1^i^	82.23 (4)	C14—C15—C16	119.01 (16)
O3—Ca1—O1^i^	97.77 (4)	C14—C15—H15	120.5
O2^i^—Ca1—O1	97.21 (4)	C16—C15—H15	120.5
O2—Ca1—O1	82.79 (4)	C17—C16—C15	121.68 (17)
O3^i^—Ca1—O1	97.77 (4)	C17—C16—H16	119.2
O3—Ca1—O1	82.23 (4)	C15—C16—H16	119.2
O1^i^—Ca1—O1	180.00 (5)	C16—C17—C18	121.11 (17)
C1—O1—Ca1	126.86 (9)	C16—C17—H17	119.4
O1—C1—C2	122.54 (14)	C18—C17—H17	119.4
O1—C1—C6	121.28 (14)	O2—C18—C17	122.19 (15)
C2—C1—C6	116.17 (13)	O2—C18—C13	121.11 (14)
C3—C2—C1	121.52 (15)	C17—C18—C13	116.68 (15)
C3—C2—H2	119.2	C18—O2—Ca1	137.34 (10)
C1—C2—H2	119.2	N2—C19—C20	111.75 (13)
C2—C3—C4	121.61 (16)	N2—C19—H19A	109.3
C2—C3—H3	119.2	C20—C19—H19A	109.3
C4—C3—H3	119.2	N2—C19—H19B	109.3
C5—C4—C3	118.83 (16)	C20—C19—H19B	109.3
C5—C4—H4	120.6	H19A—C19—H19B	107.9
C3—C4—H4	120.6	N4—C20—C19	110.89 (13)
C4—C5—C6	121.31 (16)	N4—C20—H20A	109.5
C4—C5—H5	119.3	C19—C20—H20A	109.5
C6—C5—H5	119.3	N4—C20—H20B	109.5
C7—C6—C5	117.89 (14)	C19—C20—H20B	109.5
C7—C6—C1	121.57 (14)	H20A—C20—H20B	108.0
C5—C6—C1	120.51 (14)	C21—N4—C20	122.02 (14)
N1—C7—C6	124.81 (14)	C21—N4—H4A	115.1 (13)
N1—C7—H7	117.6	C20—N4—H4A	122.6 (14)
C6—C7—H7	117.6	N4—C21—C22	125.08 (14)
C7—N1—C8	123.40 (14)	N4—C21—H21	117.5
C7—N1—H1A	115.1 (15)	C22—C21—H21	117.5
C8—N1—H1A	121.5 (15)	C21—C22—C23	118.28 (14)
N1—C8—C9	109.29 (13)	C21—C22—C27	120.90 (14)
N1—C8—H8A	109.8	C23—C22—C27	120.76 (14)
C9—C8—H8A	109.8	C24—C23—C22	120.93 (15)
N1—C8—H8B	109.8	C24—C23—H23	119.5
C9—C8—H8B	109.8	C22—C23—H23	119.5
H8A—C8—H8B	108.3	C23—C24—C25	119.02 (16)
N2—C9—C8	110.25 (13)	C23—C24—H24	120.5
N2—C9—H9A	109.6	C25—C24—H24	120.5
C8—C9—H9A	109.6	C26—C25—C24	121.57 (16)
N2—C9—H9B	109.6	C26—C25—H25	119.2
C8—C9—H9B	109.6	C24—C25—H25	119.2
H9A—C9—H9B	108.1	C25—C26—C27	121.68 (16)
C19—N2—C9	112.74 (12)	C25—C26—H26	119.2
C19—N2—C10	112.92 (12)	C27—C26—H26	119.2
C9—N2—C10	111.41 (12)	O3—C27—C26	122.55 (14)
N2—C10—C11	111.72 (13)	O3—C27—C22	121.43 (14)
N2—C10—H10A	109.3	C26—C27—C22	116.02 (14)
C11—C10—H10A	109.3	C27—O3—Ca1	150.62 (10)
N2—C10—H10B	109.3	N5—C28—C29	178.9 (3)
C11—C10—H10B	109.3	C28—C29—H29A	109.5
H10A—C10—H10B	107.9	C28—C29—H29B	109.5
N3—C11—C10	110.31 (13)	H29A—C29—H29B	109.5
N3—C11—H11A	109.6	C28—C29—H29C	109.5
C10—C11—H11A	109.6	H29A—C29—H29C	109.5
N3—C11—H11B	109.6	H29B—C29—H29C	109.5
C10—C11—H11B	109.6	O7—Cl1—O4	109.43 (13)
H11A—C11—H11B	108.1	O7—Cl1—O6	109.65 (12)
C12—N3—C11	123.08 (15)	O4—Cl1—O6	111.50 (14)
C12—N3—H3A	113.4 (14)	O7—Cl1—O5	109.34 (11)
C11—N3—H3A	123.3 (14)	O4—Cl1—O5	109.00 (12)
N3—C12—C13	124.35 (15)	O6—Cl1—O5	107.88 (10)

Symmetry codes: (i) −*x*+1, −*y*, −*z*+1.

### Hydrogen-bond geometry (Å, °)

**Table:**

Cg1 is the centroid of the C1–C6 ring.

**Table:**

*D*—H···*A*	*D*—H	H···*A*	*D*···*A*	*D*—H···*A*
N1—H1A···O1	0.85 (2)	1.98 (2)	2.6597 (17)	136 (2)
N3—H3A···O2	0.86 (2)	1.91 (2)	2.6256 (18)	140 (2)
N4—H4A···O3	0.83 (2)	1.98 (2)	2.6449 (17)	137 (2)
C3—H3···O7^ii^	0.95	2.41	3.316 (2)	160
C20—H20A···O5^iii^	0.99	2.50	3.380 (2)	148
C10—H10A···O5^iv^	0.99	2.50	3.271 (2)	134
C21—H21···O5^iii^	0.95	2.58	3.259 (2)	129
C29—H29B···O6^v^	0.98	2.58	3.555 (3)	173
C26—H26···Cg1^i^	0.95	2.68	3.489 (2)	143

Symmetry codes: (ii) −*x*+1, −*y*+1, −*z*+1; (iii) *x*−1, *y*−1, *z*; (iv) *x*−1/2, *y*−1, −*z*+3/2; (v) −*x*+3/2, *y*−1/2, *z*; (i) −*x*+1, −*y*, −*z*+1.
